# Associations between Sleep Duration and Autonomic Nervous System Regulation in Patients with Probable Alzheimer’s Disease: A Cross-Sectional Pilot Study

**DOI:** 10.3390/clockssleep6040035

**Published:** 2024-09-24

**Authors:** Chuen-Ru Liu, Chih-Yuan Yang, Dipanshu Sharma, Tun-Hao Chen, Xian-Qing Huang, Tsui-Mei Hung, Terry B. J. Kuo, Jwo-Huei Jou

**Affiliations:** 1 Taipei City Hospital Songde Branch, Taipei City 110204, Taiwan; iqlulu@mail2000.com.tw (C.-R.L.); b1512@tpech.gov.tw (T.-M.H.); 2 Institute of Brain Science, National Yang Ming Chiao Tung University, Taipei City 112304, Taiwan; albert.yc32@gmail.com; 3 Department of Materials Science and Engineering, National Tsing Hua University, Hsinchu 300044, Taiwan; dipanshusharma7374@gmail.com (D.S.); andy25512@gmail.com (T.-H.C.); sid6011000@gmail.com (X.-Q.H.)

**Keywords:** Alzheimer’s disease, autonomic nervous system regulation, dementia severity, heart rate variability, sleep duration

## Abstract

In this study, we aimed to investigate the relationships between sleep duration and autonomic nervous system (ANS) regulation. This cross-sectional pilot study included 27 older patients with probable Alzheimer’s disease who were hospitalized at a psychiatric center. We measured heart rate variability to assess ANS regulation at night, evaluated dementia severity via the Clinical Dementia Rating scale, and obtained sleep duration data from sleep diaries maintained by psychiatric nurses. The data were analyzed using repeated-measures generalized linear models with age, sex, dementia severity, hypertension status, and medication use (antipsychotics) as covariates. A sleep duration of 6–9 h per night compared to shorter than 6 h was associated with a greater increase in parasympathetic nervous system activity (*p* = 0.03), and a sleep duration longer than 9 h was associated with a decrease sympathovagal balance (*p* = 0.02). In addition, we observed an inverted U-shaped association between sleep duration and ANS regulation. In this pilot study, we demonstrated that a sleep duration of 6–9 h per night may be beneficial for ANS regulation; however, the present study involved only a few participants and had some limitations. Additional research with a larger cohort is needed to confirm these findings.

## 1. Introduction

Alzheimer’s disease (AD) is becoming increasingly prevalent, leading to a significant increase in medical expenses [1]. Among the neurodegenerative diseases, AD is common among older individuals [2]. Consequently, health concerns associated with AD have become a top priority in national public health discussions.

The autonomic nervous system (ANS) plays a crucial role during sleep and is divided into the following two branches: the sympathetic nervous system (SNS) and the parasympathetic nervous system (PSNS). Sleep consists of the following two distinct states: non-rapid eye movement (NREM) and rapid eye movement (REM). During NREM sleep, ANS activity is characterized by lower sympathetic activity and higher PSNS activity. In contrast, REM sleep is characterized by more variable ANS activity, with periods of both SNS and PSNS influences [3,4,5].

Sleep disturbances and delayed circadian rhythm are common symptoms of AD, and they have generally been considered as late consequences of the neurodegenerative processes that affect a significant proportion of people with AD, ranging from 45% to 61.3% [6,7]. Adequate sleep duration is pivotal for mitigating psychological symptoms and delaying AD-related decline [8,9]. AD reduces cholinergic activity and cerebral blood flow, perpetuating chronic vasoconstriction and reducing cerebral perfusion, thus fostering the accumulation of amyloid-beta and tau proteins and impairing neurological function [10]. Sleep deprivation, prolonged sleep duration, and circadian rhythm delay are associated with neurodegenerative changes. Amyloid plaques are linked to synaptic loss, neuronal death, and cognitive impairment in AD, primarily involving the accumulation of β-amyloid protein (Aβ), neurofibrillary tangles (NFTs), neuroinflammation, oxidative stress, circadian rhythm dysfunction, and reduced aldehyde dehydrogenase 2 (ALDH2) enzyme activity. These factors may exacerbate monoaminergic neuronal loss and synucleinopathies, which may be related to the degradation of brain regions controlling ANS functions [11,12,13].

ANS dysregulation can lead to sleep disturbances [2,10]. ANS regulation serves as an index of PSNS activation and sympathetic nervous system (SNS) deactivation during the night. While both the PSNS and SNS innervate key control points of cerebral perfusion, PSNS fibers strongly innervate the cerebral vasculature, resulting in cerebral vasodilation. Consequently, PSNS input enhances cerebral perfusion, whereas SNS input diminishes cerebral perfusion during sleep [10]. The PSNS acts as a “brake” on the SNS [6,14,15,16,17]. PSNS activity decreases with age, particularly in individuals with AD, resulting in progressive ANS dysregulation [15,18,19,20]. In patients with all types of dementia, particularly in the case of AD, PSNS activity during both NREM and REM sleep stages is lower than that in healthy individuals. This phenomenon may be related to the degeneration of brain regions that control the ANS [3,4]. A sleep duration that exceeds 9 h or is less than 6 h disrupts ANS regulation, contributing to bidirectional dysregulation, thereby destabilizing physiological processes, and contributing to various pathologies, such as cognitive impairment, metabolic disorders, oxidative stress, inflammation, immune dysregulation, cardiovascular disease, coronary heart disease, cancer, and mental disorders [2,7,21,22,23,24,25,26]. ANS dysregulation, characterized by increased SNS activity, reduces cerebral blood flow, which exacerbates AD-related symptoms, such as deficits in hippocampal synaptic plasticity, decreased slow-wave sleep (SWS), restless leg syndrome (RLS), periodic limb movement disorder (PLMD), and REM sleep behavior disorder (RBD), and inhibits acetylcholine secretion [17,27,28,29]. Triggering SNS activity increases baroreceptor and catecholamine levels, leading to a rapid transition to permissive tachycardia, which plays a crucial role in diseases associated with cardiovascular alterations, obstructive sleep apnea (OSA), and hypertension [30]. Acetylcholine neurotransmitter levels are positively correlated with PSNS activity, suggesting that reduced night-time PSNS activity serves as a key indicator of decreased dementia severity [6,14,31,32]. Higher PSNS activation at night may indicate better ANS regulation [15,33,34]. Reduced sleep duration and SWS activity impede frontal lobe restoration, while interventions targeting sleep quality aim to facilitate amyloid-beta clearance and promote SWS, with a focus on memory encoding and consolidation [29]. This pilot study suggests that 6–9 h of sleep per night are defined as adequate durations.

The ANS includes the SNS and the PSNS, the activity of which can be assessed by heart rate variability (HRV) [3,16,33,35]. HRV refers to the complex variation in heart rate resulting from the interaction between the SNS and PSNS (vagal) neural activity at the sinus node [3,15,16,30,36]. Noninvasive HRV measurements are utilized in sleep and ANS research. Sympathovagal balance, reflecting the modulation of the SNS and PSNS, is crucial during sleep, particularly with respect to ANS dysregulation in AD patients [17]. Discoordination of SNS and PSNS activities in individuals with AD, resulting in ANS dysregulation, appears to be associated with extreme sleep durations. Therefore, in our study, we aimed to investigate the relationships between sleep duration and ANS regulation. We hypothesized that an adequate sleep duration would exert beneficial effects on nocturnal ANS regulation.

## 2. Results

### 2.1. Demographic and Clinical Characteristics

Twenty-seven participants completed the study successfully. Each participant underwent 9 h of HRV measurements over one day, resulting in 27 datasets for analysis. Notably, no significant differences in clinicodemographic characteristics, such as sex (χ^2^ test = 1.0, *p* = 0.58), hypertension (χ^2^ test = 1.1, *p* = 0.56), or probable AD severity (χ^2^ test = 6.9, *p* = 0.86), were observed among the five groups. Overall, 59% of the participants were female, 41% were male, and approximately 45%, 44%, and 11% had mild, moderate, or severe probable AD, respectively. The sleep duration was distributed as follows: <6 h, 29.6% of the participants; 6–9 h, 44.4% of the participants; >9 h, 25.9% of the participants.

To assess the impact of comorbidities, including diabetes, congestive heart failure, and cerebrovascular disease, on sleep duration, the Kruskal–Wallis test was used to analyze continuous variables such as age, comorbidity index, and medication dosage. No significant differences were found among the groups, underscoring the homogeneity of the participant distribution.

The mean age was 79.5 years (SD = 8.5 y). Antipsychotic medication was administered to 93% of the respondents, with a mean medication-defined daily dose (DDD) of 0.86 (SD = 1.34). In addition, 78% of participants used antidepressants at a mean dose of 0.62 (SD = 0.65), whereas 81% used benzodiazepines at a mean dose of 0.26 (SD = 0.26). Participants with a sleep duration exceeding 9 h consumed a higher average dose of antipsychotic medication (1.3 units) compared to those in other groups. The results of the descriptive statistical analysis of all the variables are shown in Table 1. The study also evaluated the effect of age on PSNS activity, with mean values of 5.5, 3.5, 4.3, and 6.3 for the age groups 60–69 y, 70–79 y, 80–89 y, and 90–99 y, respectively. No decrease in PSNS activity with increasing age was observed.

### 2.2. Outcomes

#### 2.2.1. Effect of Short Sleep Duration on ANS Regulation

The ANS, encompassing the SNS and PSNS, regulates involuntary bodily functions. HRV measures the temporal variation between heartbeats, with LF and HF power reflecting the activity levels of the SNS and PSNS, respectively. The LF/HF ratio indicates the balance between these systems, where a higher ratio suggests increased SNS activity, which is associated with elevated mortality and major cardiovascular risks, while a lower ratio reflects a more balanced or PSNS-dominant state, associated with improved regulatory and relaxation outcomes. The data were analyzed using repeated-measures generalized linear models with age, sex, dementia severity, hypertension status, and medication use (antipsychotics) as covariates.

**Figure 1:**
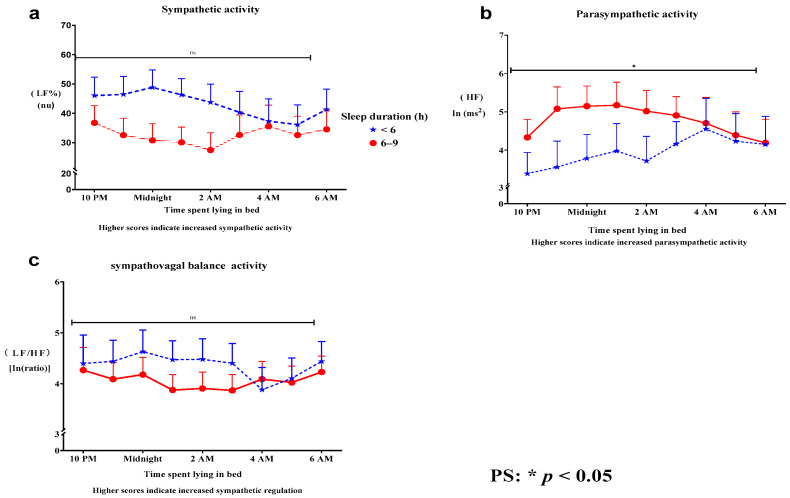
A sleep duration of 6–9 h per night was associated with better ANS regulation than a sleep duration of less than 6 h. Specifically, 6–9 h of sleep per night was associated with the following trends: (**a**) decreased SNS activity, (**b**) increased PSNS activity, and (**c**) a decrease in sympathovagal balance (**a**–**c**). Additionally, a sleep duration of 6–9 h per night was associated with a significant increase in PSNS activity (**b**). No significant differences were observed in mean SNS activity (**a**) or sympathovagal balance (**c**). ANS, autonomic nervous system; HF, high-frequency power, reflecting PSNS activity; LF%, normalized low-frequency power, reflecting SNS activity; LF/HF, low-frequency/high-frequency ratio, reflecting sympathovagal balance (SNS regulation); SNS, sympathetic nervous system; PSNS, parasympathetic nervous system.

Psychiatric nurses monitored eye movement, depth of breathing, and frequency of body movement. Participants with a sleep duration of 6–9 h per night exhibited enhanced ANS regulation compared to those with less than 6 h of sleep. Specifically, a sleep duration of 6–9 h was associated with (a) decreased SNS activity, (b) increased PSNS activity, and (c) a reduced sympathovagal balance (Figure 1a–c) during the bedtime period from 9:00 p.m. to 6:00 a.m. Moreover, compared to individuals with shorter sleep durations, those with 6–9 h of sleep per night showed a significant increase in PSNS activity (Roy’s largest root = 5.74, *p* = 0.03) (Figure 1b). In this group, PSNS activity peaked earlier and demonstrated a greater phase increase compared to other sleep durations. In contrast, sleep durations shorter than 6 h were associated with impaired PSNS activity (Figure 1b and Table 2). No significant differences were observed in the mean SNS activity (Roy’s largest root = 0.15, *p* = 0.86) (Figure 1a) or sympathovagal balance (Roy’s largest root = 1.02, *p* = 0.5) (Figure 1c).

In summary, the study suggests that a sleep duration of 6–9 h is linked to a significant reduction in sympathovagal balance at 11 p.m., with a sustained peak in PSNS activity noted at 1 a.m.

#### 2.2.2. Effect of Longer Sleep Duration on ANS Regulation

**Figure 2:**
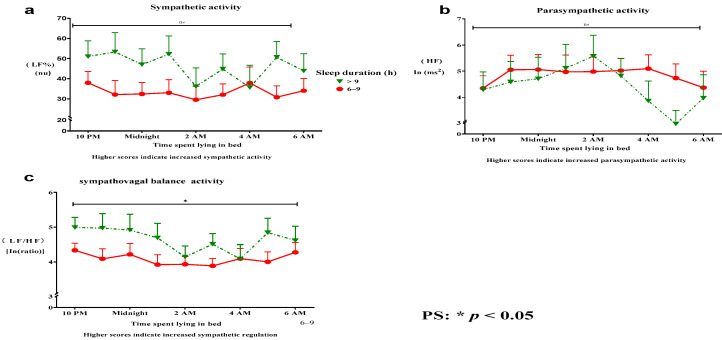
A sleep duration of 6–9 h per night was associated with better ANS regulation than a sleep duration longer than 9 h. A sleep duration of 6–9 h per night was associated with the following trends: (**a**) decreased SNS activity, (**b**) increased PSNS activity, and (**c**) a decrease in sympathovagal balance (**a**–**c**). In addition, a sleep duration of 6–9 h per night was associated with a significant decrease in sympathovagal balance (**c**). (**c**) No significant differences in mean SNS activity (**a**) and PSNS activity (**b**) were observed. ANS, autonomic nervous system; HF, high-frequency power, reflecting PSNS activity; LF%, normalized low-frequency power, reflecting SNS activity; LF/HF, low-frequency/high-frequency ratio, reflecting sympathovagal balance (SNS regulation); SNS, sympathetic nervous system; PSNS, parasympathetic nervous system.

Compared with participants with longer sleep durations, participants with a sleep duration of 6–9 h per night demonstrated increased ANS regulation. A sleep duration of 6–9 h per night was associated with the following trends: (a) decreased SNS activity, (b) increased PSNS activity, and (c) a decrease in sympathovagal balance (Figure 2a–c) during the bedtime period from 9:00 p.m. to 6:00 a.m. In addition, a sleep duration of 6–9 h per night compared with a sleep duration longer than 9 h was associated with a decrease in sympathovagal balance (Roy’s largest root = 10.37, *p* = 0.02) (Figure 2c).

Conversely, sleep durations > 9 h were associated with increased PSNS activity, which was associated with significant periodic fluctuations and a lack of regular nocturnal rhythms (Figure 2b). Thus, an inverted U-shaped association between sleep duration and ANS regulation was identified. A longer sleep duration was associated with more severe impairment of ANS regulation than a sleep duration of 6–9 h (Table 2). Non-significant differences in the mean SNS activity (Roy’s largest root = 0.63, *p* = 0.72) (Figure 2a) and PSNS activity (Roy’s largest root = 0.53, *p* = 0.78) (Figure 2b) were detected.

## 3. Discussion

In this pilot study, we assessed sleep duration based on psychiatric nurse observations of eye movement, depth of breathing, and body movement frequency in patients with AD. Our findings indicate that a sleep duration of 6–9 h per night was associated with improved ANS regulation, evidenced by increased PSNS activity and decreased SNS activity compared to those of other sleep durations, such as sleep durations of <6 h and >9 h. Compared to an adequate sleep duration, a sleep duration longer than 9 h was associated with a more severe impairment of sympathovagal balance. We found that longer sleep durations were associated with more severe impairment of SNS activity and sympathovagal balance, possibly for the following reasons: (1) excessive use of sleeping drugs, leading to drowsiness; (2) poor physical fitness, requiring more time to rest; (3) severe dementia. The authors of this study speculated that a longer sleep duration can affect ANS regulation and may result in periodic fluctuations (a rapid rise and fall), resulting in the lack of regular nocturnal rhythms.

Compared with other sleep durations, a sleep duration of 6–9 h tended to be associated with increased PSNS activity, decreased SNS activity, and decreased sympathovagal balance. A sleep duration of 6–9 h per night was associated with increased PSNS activity compared with a sleep duration shorter than 6 h and a decrease in sympathovagal balance compared with a sleep duration longer than 9 h; the association between sleep duration and ANS regulation was characterized by an inverted U-shaped curve.

We found that sleeping 6–9 h per night was associated with better ANS regulation than sleeping for other durations. These findings were consistent with those of previous studies [12,17,22,26,37,38,39] showing that longer or shorter sleep durations can cause ANS dysregulation [17,20,30,34,40]. Monitoring HRV during sleep may be more accurate and reliable for measuring ANS activity, considering there is less influence from factors such as physical activity, stress, and emotions, which may mask or confound the underlying patterns of ANS activity [4].

### 3.1. ANS Dysregulation Is Related to Inadequate Sleep Duration in Sympathetic-Mediated Diseases

The flip–flop switch model of sleep–wake regulation suggests that sleep-inducing neurotransmitters, such as gamma-aminobutyric acid and the neuropeptide galanin, are located in the ventrolateral preoptic nucleus of the hypothalamus. The release of these neurotransmitters promotes sleep. Conversely, wakefulness begins with inhibition of the ventrolateral preoptic nucleus involving cholinergic, monoaminergic, and orexinergic neurons. The sleep–wake system is a critical regulator of homeostasis [16,30,40]. An imbalance in the ANS can lead to sleep disturbances.

Inadequate sleep duration is associated with ANS dysregulation and contributes to several sympathetic-mediated diseases, including increased levels of proinflammatory cytokines such as interleukin-6, high-sensitivity C-reactive protein, and tumor necrosis factor-alpha. It also disrupts the cyclic adenosine monophosphate and gamma-aminobutyric acid signaling pathways, affecting synaptic plasticity. Altered melatonin secretion may promote tumor growth, whereas gray matter volume loss may occur in cognitive regions. In addition, increased levels of glucocorticoids and reactive oxygen species can lead to DNA damage. Sleep deprivation can exacerbate synucleinopathies, increase cerebrospinal fluid levels of amyloid-beta 42 and tau peptides, and decrease ALDH2 enzyme activity, leading to neurotoxic aldehyde accumulation and neuronal degeneration [4,11,36,39,41].

Our results suggest that extremely short and long sleep durations may contribute to ANS dysregulation. Such impairments are reflected by an increase in ANS activity and an altered sympathovagal balance like that observed in patients with probable AD. We speculate that inadequate sleep duration may induce a chronic state of vasoconstriction, leading to reduced cerebral perfusion, a potential marker of impaired autonomic tone, particularly excessive SNS activity [10]. This finding is consistent with the findings of a previous study by Wang et al. (2019) [42], who reported increased mortality and major cardiovascular risks associated with both longer and shorter sleep durations.

### 3.2. Medications Disrupt Bidirectional Associations between Sleep Duration and ANS Regulation

ANS regulation plays an important role in the wake-to-sleep transition through cardiorespiratory and brain regulation [43], especially for ANS dysregulation in patients with AD [17].

In the present study, a sleep duration of 6–9 h was associated with a significant decrease in sympathovagal balance during 1 h of sleep (11 p.m.). In contrast, a sustained peak in PSNS activity was observed at 1 a.m. Healthy individuals exhibited maximal PSNS activity at 2 a.m. [44]. There is lower PSNS activity with insomnia (5.6 versus 4.0) [45], and greater sympathovagal balance in older, healthy individuals (1.1 versus 4.3) [46]. Compared with those in older healthy individuals with insomnia, PSNS activity in indi-viduals with AD peaks and exhibits a low-phase shift, resulting in decreased PSNS activity. Therefore, the underlying mechanism through which increasing PSNS activity promotes sleep and results in physical and mental relaxation differs from that through which sleep medications modulate decreased SNS activity and induce a longer sleep duration. The PSNS is predominantly regulated by the vagus nerve and can inhibit muscle activity, reducing the occurrence of PLMD and RBD during phasic REM sleep [18,20,30,47].

Sleep medications commonly used to promote sleep include antidepressants, antipsychotics, benzodiazepines, and acetylcholinesterase inhibitors [28,42,48]. These drugs affect ANS regulation by exerting neuroleptic effects and blocking SNS receptors. Antipsychotics with strong anticholinergic properties are associated with a reduction in SNS and PSNS activity [49,50]. The participants in the >9-h sleep duration group consumed a greater average dose of antipsychotic medication than those in the other groups.

Quetiapine is the most prescribed antipsychotic drug for treating dementia in Taiwan. Huang’s study (2016) demonstrated that quetiapine significantly reduces activity in both the SNS and PSNS [50]. It is inferred that this medication likely has a negative effect on sleep quality. Furthermore, through medication, altered sympathovagal balance was observed, as noted in this study. This disrupts ANS regulation and increases cardiovascular risk. Sleep disturbances can lead to neurodegeneration through products that not only affect disease progression but also cause cognitive impairment. Additionally, sleep disturbances may be considered a marker of neurodegeneration [4].

In the 6 to 9-h sleep group of individuals with AD, PSNS activity peaks early and exhibits a greater phase increase than other sleep durations. Therefore, the underlying mechanism by which increasing PSNS activity promotes sleep and achieves physical and mental relaxation differs from that by which sleep medications modulate SNS activity and induce sleep.

### 3.3. Sleep Duration Effects on PSNS Activity

ANS regulation plays a crucial role in adjusting the pacemaker rate to modulate sleep–wake cycles and nocturnal rhythms, thereby maintaining homeostasis during the integration of neuroendocrine systems [40]. Our study indicates that sleep duration significantly influences nocturnal ANS regulation. Specifically, sleep durations of less than 6 h were associated with more pronounced impairment in PSNS activity compared to adequate sleep durations.

In contrast to the literature suggesting a decline in PSNS activity with aging in healthy individuals [18,19,20], our findings did not demonstrate a decrease in PSNS activity attributable to age. Given that night-time PSNS activity is a critical predictive factor for sleep-dependent cognitive function [16], we presume that maintaining an adequate sleep duration may mitigate the age-related decline in PSNS activity.

### 3.4. Feasibility of This Study

The success rate for wearing an HRV device was 45%. The patients reported that the device felt like a foreign body when worn on the chest, and it was easy for them to remove it by themselves. To gain better trust from patients, gaming interactions (such as talking and singing) were introduced in this study and are recommended in similar studies in the future. To further increase adaptability, wearable devices are also recommended for real-time HRV monitoring.

The measurements of sleep duration used in the current study may have introduced subjectivity. Future studies should employ more objective sleep assessment methods, such as polysomnography.

### 3.5. Limitations

This study had several limitations. First, we used a cross-sectional survey conducted as a pilot study, which may limit the generalizability of the findings. Second, this study involved longitudinal and continuous monitoring of night-time HRV in a specific group of participants with probable AD. However, the results are limited by the difficulty in obtaining participants and the relatively small sample size. Third, only participants with probable AD were included, but the findings can also be used as a reference for patients with other types of dementia. Fourth, sleep diary sleep assessment data need to be more objective. Fifth, many interfering factors affect ANS regulation, and it is difficult to comprehensively consider them; this study did not control for many variables, including OSA, beta-blocker medication, RLS, PLMD, or RBD, which are known to affect ANS regulation. The design of this study controlled for only age, sex, hypertension status, dementia severity, and medication (antipsychotics) use as covariate variables. Finally, in this study, dementia severity was assessed by direct scoring of the CDR as a secondary medical record. The CDR is a multifaceted assessment tool that includes six aspects and is evaluated by professional psychologists, doctors, and nurses; therefore, it is still highly objective.

## 4. Materials and Methods

### 4.1. Ethics Statement

The research protocol (TCHIRB-10705110) was reviewed and approved by the institutional review board. Prior to participation, both the participants and their caregivers provided written informed consent.

### 4.2. Study Design

This was a measurement-based cross-sectional survey conducted as a pilot study. The study was conducted between 2018 and 2020 among 35 patients with probable AD who were hospitalized at a psychiatric center in Taipei, Taiwan. Dementia of the AD type requires a history of cognitive decline and impairment in activities of daily living, con-firmed by a neurologist and documented in medical records. The primary objective was to measure HRV to quantify ANS regulation during the night. The participants’ behavioral and psychological symptoms of dementia typically led to caregiver burden and hospitalization, with an average length of stay of 40 days. Behavioral and psychological symptoms were relatively stable after 14 days of hospitalization. The participants showed no aggressive or disruptive behavior, and they were still in bed at night, which prevented them from removing and destroying the equipment at night (according to the participants’ caregivers, who insisted on being with them during the HRV wearing time). To acclimate participants to the feeling of the HRV device on their body, it was recommended that they try it between 5:00 p.m. and 6:00 p.m. for 1–5 days until they were willing and able to complete 1 day of 9 h (9:00 p.m. to 6:00 a.m.) of HRV measurement with the wearable device.

The participants’ bedtimes were based on hospital routines, with bedtime medications administered between 8:00 p.m. and 8:30 p.m., and lights turned off at 9:00 p.m., with the participants lying in bed. Throughout the night, psychiatric nurses observed eye movement, depth of breathing, and frequency of body movements to determine the differences between falling asleep and waking. Sleep duration refers to the total amount of sleep obtained during the period between falling asleep and waking up, excluding all periods of wakefulness. The participants were divided into the following three groups on the basis of their reported sleep duration: <6 h, 6–9 h, and >9 h. Dementia severity was assessed by direct scoring of the CDR as a secondary medical record and was categorized into the following three levels: mild, moderate, and severe. The participants were divided into the following four age groups: 60–69 y, 70–79 y, 80–89 y, and 90–99 y, and into hypertension and no-hypertension groups.

### 4.3. Participants

The required sample size was calculated using G Power 3.1 computer software, which yielded an estimate of 20 participants. The data were analyzed using analysis of variance SPSS (G*Power 3.1). Statistical significance was indicated by α = 0.05 and β = 0.2, with an effect size of 0.3 [51]. F tests and repeated-measures ANOVA were used to examine within-between interactions. The starting sample size was 30 participants across three groups.

Sleep duration, measured from sleep onset to wake onset (09:00 p.m. to 06:00 a.m.), was quantified for each night using sleep diaries. The mean sleep duration over the 5-day observation period was determined for each participant. For the final analysis, one day with at least 9 h (09:00 p.m. until 6:00 a.m.) of HRV measurements was required to assess ANS regulation at night.

The inclusion criteria for participants were as follows: (1) met the standard clinical criteria for probable AD according to the International Classification of Diseases, 10th Revision, Clinical Modification [52], as diagnosed by specialists based on the course of the disease or other reports; (2) were acutely hospitalized with probable AD, defined as memory and cognitive impairment, and classified as mild, moderate, or severe AD; (3) were aged ≥ 60 years; (4) voluntarily participated in the study (both participants and their caregivers); (5) were willing to use an HRV instrument at the end of the study to complete 1 day of 9-h HRV measurements. The exclusion criteria were as follows: (1) the presence of a pacemaker or a medical history of arrhythmia [53], as reported by caregivers and recorded in medical records; (2) other diseases that affect sleep, such as delirium, fever, or unstable vital signs.

Among the 35 patients eligible for inclusion in the study, eight discontinued because they were unable to complete one day of 9-h HRV measurements. Consequently, only 27 patients successfully completed the study.

### 4.4. Instruments and Outcome Measures

The primary objective was to assess the effects of sleep duration on nocturnal ANS performance and dementia severity.

We determined the primary outcome measures, including the mean values of the trial variables, such as HF power, LF power, and the LF/HF ratio, from the HRV data. PSNS activity was indicated by the HF component, SNS activity was indicated by LF%, and sympathovagal balance was indicated by the LF/HF ratio, reflecting the modulation of SNS activity. Dementia severity was the secondary outcome, as assessed by the Washington University Clinical Dementia Rating (CDR) score. The CDR rates an individual’s dementia severity in the following six domains: memory, orientation, judgment, problem-solving, community affairs, home, and hobbies. The CDR score indicates mild (0.5–1), moderate (2), or severe (3) dementia [54].

#### 4.4.1. Validity and Reliability of the HRV Data

We evaluated one component each in the LF (0.04–0.15 Hz) and HF (0.15–0.40 Hz) ranges. The LF/HF ratio is negatively correlated with delta power (0.5–4.0 Hz), which is measured by electroencephalography during quiet sleep, whereas cardiac sympathovagal balance is negatively correlated with sleep depth [16,54,55,56].

The precordial electrocardiogram (ECG) recordings were obtained from 9:00 p.m. to 6:00 a.m. while participants remained at rest. The ECG data were collected using a standard HRV device, model WG-103A, with monitoring occurring every 5 min throughout the entire 9-h duration of the night.

The electrodes for the ECG placement were positioned over the sinus node to ensure continuous recording during overnight bed rest. The data were collected at 2.5-min intervals over the course of a single day, constituting 9 h of HRV measurements. Analysis was conducted using the KY laboratory software package, accessible at http://xds3.cmbm.idv.tw/sl [57]. We adjusted the HRV parameters for confounders, system settings, and statistical settings, and excluded extreme values. The real-time signal was transmitted to a smartphone using a Xenon Bluetooth Low Energy as a low-cost and high-efficiency measurement tool. HRV data were sent to the respective sensor and mobile device to achieve high validity and reliability.

#### 4.4.2. Validity and Reliability of Sleep Duration Data

A sleep diary is a standard assessment instrument used in psychiatric hospital routines that allows for the prospective monitoring of sleep patterns in the hospital. The variables derived from sleep diaries included sleep-onset latency and wake time after sleep onset. Sleep duration refers to the total amount of sleep obtained during the period between sleep-on and sleep-off, excluding of the awake stages [45].

Each night, two psychiatric nurses took turns visiting the participants every 30 min from 9:00 p.m. to 6:00 a.m. to complete the sleep diaries. The sleep diaries were based on observations of voluntary eye opening, breathing depth, and limb movement frequency. Each psychiatric professional received training prior to performing the sleep diary observation technique. The psychiatric nurses completed the sleep diaries with 80% consistency and accuracy.

### 4.5. Statistical Analyses

The demographic characteristics are summarized as frequencies and percentages (*n*, %) for categorical variables and means for continuous variables. The demographic characteristics of the three sleep duration groups were compared via the chi-square test. We used repeated-measures generalized linear models (GLMs) to examine the correlations of sleep duration and ANS regulation with age, sex, dementia severity, and medication (antipsychotics) as covariates, as well as the correlations of dementia severity and ANS regulation with age, sex, hypertension status, and medication (antipsychotics) as covariates. Analyses were performed using SPSS 24 software, and a *p*-value < 0.05 indicated statistical significance. We examined the effects of sleep duration on ANS regulation by using indicators of SNS activity, sympathovagal balance (LF% and LF/HF ratio), and PSNS activity (HF power component from HRV data). We also analyzed the main effects of different sleep durations (6–9, >9, and <6 h) on ANS regulation.

## 5. Conclusions

In this cross-sectional pilot study, we demonstrated that a sleep duration of 6–9 h per night may be beneficial for ANS regulation. A sufficient sleep duration may lead to better ANS regulation. Following the recommendation to maintain a sufficient sleep duration of 6–9 h per night may result in increased night-time PSNS activity, reduced SNS activity, and thereby the alleviation of cognitive impairment; however, the present study involved only a few participants and had some limitations. Additional research with a larger cohort is needed to confirm these findings.

## Figures and Tables

**Table 1:** Participant characteristics.

Characteristic	Sleep Duration < 6 h	Sleep Duration of 6–9 h	Sleep Duration > 9 h	Total	*p*-Value ^a^
Sex					0.58
1. Male	3 (37.5%)	4 (33.3%)	4 (57.1%)	11 (40.7%)	
2. Female	5 (62.5%)	8 (66.7%)	3 (42.9%)	16 (59.3%)	
Severity of AD					0.86
1. Mild	5 (50%)	5 (41.7%)	2 (14.3%)	12 (44.4%)	
2. Moderate	2 (25%)	6 (50%)	4 (57.1%)	12 (44.4%)	
3. Severe	1 (12.5%)	1 (8.3%)	1 (14.3%)	3 (11.1%)	
Hypertension status					0.56
Yes	4 (50.0%)	8 (66.7%)	3 (42.9%)	15 (55.6%)	
No	4 (50.0%)	4 (33.3%)	4 (57.1%)	12 (44.4%)	
	Mean (SD)				
Age, y	81.2 ± 6.7	78.7 ± 9.4	79.3 ± 9.7	79.5 ± 8.5	0.67
Medication dosage					
Antipsychotics	0.3 ± 0.3	1.0 ± 1.3	1.3. ± 1.8	0.8 ± 1.3	0.27
Antidepressants	0.7 ± 0.7	0.5 ± 0.8	0.8 ± 0.6	0.6 ± 0.6	0.24
Benzodiazepines	0.3 ± 0.3	0.2 ± 0.2	0.2 ± 0.3	0.2 ± 0.2	0.36

^a^ The Kruskal–Wallis test was used to analyze continuous variables, whereas the chi-square test was used to analyze categorical variables. AD, Alzheimer’s disease.

**Table 2:** Generalized linear model analysis of the effects of sleep duration on ANS regulation.

Sleep Duration per Night	LF% ^a^ Mean (SE) ^d^	*p*-Value ^e^	HF ^b^ Mean (SE) ^d^	*p*-Value ^e^	LF/HF ^c^ Mean (SE) ^d^	*p*-Value ^e^
Sleep duration < 6 h ^f^	41.8 (5.2)		4.0 (0.5)		4.3 (0.3)	
Sleep duration of 6–9 h ^g^	34.0 (4.9)		4.8 (0.4)		4.1 (0.2)	
Sleep duration > 9 h ^h^	45.7 (6.9)		4.5 (0.6)		4.8 (0.3)	
Shorter sleep duration ^i^		0.86		0.03 *		0.5
Longer sleep duration ^j^		0.72		0.78		0.02 *

^a^ Normalized low-frequency power, reflecting SNS activity. ^b^ High-frequency power, reflecting PSNS activity. ^c^ Low-frequency/high-frequency ratio, reflecting sympathovagal balance (SNS regulation). ^d^ Mean/standard error: hours of sleep or dementia severity in the autonomic nervous system (lying in bed from 9:00 p.m.to 6:00 a.m.). ^e^ Roy’s largest root. ^f–h^ Sleep duration per night: total sleep time according to the sleep diary. ^i^ Shorter sleep duration: a sleep duration of 6–9 h per night compared to a sleep duration shorter than 6 h. ^j^ Longer sleep duration: a sleep duration of 6–9 h per night compared to a sleep duration exceeding 9 h. * *p* < 0.05. ANS, autonomic nervous system; HF, high-frequency power; LF%, normalized low-frequency power; LF/HF, low-frequency/high-frequency ratio; SNS, sympathetic nervous system; PSNS, parasympathetic nervous system.

## Data Availability

The data are accessible from the corresponding author upon reasonable request.
